# Proceedings of the 2013 Meeting of the Australasian Section of the American Oil Chemists Society (AAOCS)

**DOI:** 10.3390/nu5125065

**Published:** 2013-12-12

**Authors:** Karen Murphy, Peter Howe

**Affiliations:** 1 Nutritional Physiology Research Centre, University of South Australia, Adelaide, SA 5001, Australia; E-Mail: karen.murphy@unisa.edu.au; 2 Clinical Nutrition Research Centre, University of Newcastle, Callaghan, NSW 2308, Australia

## 1. Preface

The Australasian section of the American Oil Chemists Society (AAOCS) held their biennial meeting in Newcastle, Australia from 6 to 8 November, 2013. Over 150 scientists, researchers and industry representatives gathered for three days of talks and discussions on a variety of lipid related topics. The AAOCS awarded its inaugural *AAOCS Award for Scientific Excellence in Lipid Research* to Dr Allan Green from the Commonwealth Scientific and Industrial Research Organisation (CSIRO). Dr Green is deputy chief of the CSIRO Division of Plant Industry and has been active in lipid research for several decades. His main research focus is on plant breeding and genetic engineering techniques to develop improved oilseeds with enhanced human nutritional value and novel industrial uses. Refer to “*AAOCS Award for Scientific Excellence in Lipid Research*” for more detail of his contributions [1].

A highlight of the meeting was a whole day symposium on the recent advances in Omega-3 health benefits, sources, products and bioavailability which was jointly hosted by the Omega 3 Centre. Prof. Clemens von Shacky from the Preventive Cardiology unit at the Ludwig Maximilians-University of Munich opened the symposium with a thought provoking keynote address on the Omega-3 Index as a biomarker of heart health. He emphasized the importance not only of limiting intervention trials to those identified with low initial omega-3 status but also ensuring that omega-3 supplements are consumed with a meal, as bioavailability data indicated that omega-3 uptake was dependent on concomitant fat intake. Dr David Colquhoun and Michael Macartney also spoke on omega-3 and heart health, while Prof Peter Howe presented data linking the Omega-3 Index to improved body composition. Dr Bev Muhlhausler discussed the importance of omega-3 for fetal and infant development, including recent evidence which suggests a potential link between increased exposure to long chain omega-3 *in utero* and improved metabolic and cardiovascular outcomes later in childhood. A/Prof Andrew Pipingas and Dr Welma Stonehouse gave presentations on neurocognitive benefits of omega-3 in healthy adults. They were followed by A/Prof Lisa Wood and Prof Michael James who gave presentations on anti-inflammatory benefits of omega-3 in asthma and rheumatoid arthritis respectively, emphasising in each case the potential for omega-3 as adjunct therapy.

Adam Ismail from the Global Organization for EPA and DHA Omega-3(GOED) gave a very entertaining talk on the omega-3 market. Adam highlighted the growth of omega-3 in pharmacology and the challenges of meeting increasing demand with increased supply of a natural resource. This led into Dr Giovanni Turchini’s talk on the use of omega-3 in aquaculture. He noted that the majority of aquaculture (seaweed, mollusc and fresh water fish) can actually produce omega-3 as a source and he emphasised that aquaculture diets have changed to meet demand of the growing industry. Nils Hoem from Aker BioMarine, Norway described the growing krill oil market and detailed the chemistry of Antarctic krill oil. Novel oil sources such as Algal EPA, by Hywel Griffiths from Photonz, New Zealand and genetic engineered oil seed crops by Surinder Singh form CSIRO Food Futures National Research Flagship and CSIRO Plant Industry highlighted the cutting edge science being undertaken in this part of the world to meet demand for omega-3 and omega-3 products.

A well-attended workshop held in conjunction with the conference brought scientists together with industry leaders to examine the principles behind deep-frying and frying oils. A variety of applied topics of importance to food manufacturers that produce fried products were discussed, ranging from the chemistry and physics of deep frying, fryer design and novel specialised oils through to regulatory requirements.

A goal of the Australasian section of AAOCS is helping to develop future researchers and industry leaders. We had more student oral and poster presentations than ever before and they were of very high quality. AAOCS were grateful to *Nutrients* for sponsoring the student awards this year. Tim Nalder from Plant and Food Research New Zealand/ Deakin University won the Bryce Bell student prize for best oral communication describing characterisation of lipase fatty acid selectivity using novel omega-3 pNP-acyl esters. Cintia Dias from the University of Newcastle received a *Nutrients* encouragement award for her oral communication on determining how omega-3 fatty acids alleviate dietary saturated fat-induced postprandial rise in blood lipid levels. The Rod Mailer student poster prize was awarded to Clare Flakelar from the Charles Sturt University and Graham Centre for Agricultural Innovation. Clare’s work on assessing Australian canola oil for relationships between oxidative stability, trace elements and fatty acid profiles of selected cultivars will provide baseline data for development of new canola lines and further development of the canola industry. Finally the *Nutrients* encouragement poster award was won by Reinu Abraham from Deakin University for his work looking at different cellulosic biomass for the microbial production of biofuels, lipids and carotenoids. 

## 2. Summary of Scientific Presentations

### 2.1. Omega-3 Index for Heart Health—Clemens von Schacky

Recent intervention trials and pertinent meta-analyses had neutral results in terms of reductions in total mortality or adverse cardiac events by increased intake of eicosapentaenoic acid (EPA) plus docosahexaenoic acid (DHA). Safety and tolerability of EPA + DHA were identical to placebo. Cardiovascular guidelines do not uniformly recommend EPA + DHA, and many cardiologists are reluctant to advise them to their patients. The neutral results mentioned are in contrast to epidemiologic studies, generally demonstrating up to 50% lower incidence of adverse cardiac events, depending on intake of EPA + DHA, and in even sharper contrast to epidemiologic studies based on levels of EPA + DHA in red blood cells, demonstrating e.g. a 10-fold lower incidence of sudden cardiac death, 4-fold lower incidence of total mortality, and 3-fold lower incidence of non-fatal acute coronary syndrome. EPA + DHA in red blood cells, when measured with a proprietary standardized analytical procedure conforming to the rules of Clinical Chemistry, is called HS-Omega-3 Index. A low HS-Omega-3 Index is a cardiovascular risk factor (by American Heart Association criteria), and has a causal role in impairments in cognitive and other brain functions. In all populations studied so far, the HS-Omega-3 Index had a statistically normal distribution; means, however, varied substantially from population to population. Bioavailability of EPA + DHA depended on fat content of a concomitant meal (if any), emulsification, dietary source (fish, fish oil, krill oil, microalgal oil), chemical form, and individual and other factors. Consequently, the relationship between intake of EPA + DHA and the HS-Omega-3 Index is loose. In the intervention trials mentioned, participants had been recruited irrespective of their baseline Omega-3 Index, and exposed to identical doses of EPA + DHA. Therefore, levels of EPA + DHA overlapped between active and control (placebo) groups, compromising their differentiation, thereby substantially increasing the chance of neutral results. In the future, study participants with a low Omega-3 Index should be recruited and exposed to individually tailored doses to reach a target HS-Omega-3 Index of 8%–11%. This will lead to clearer results and probably to less contrast between epidemiologic and intervention studies. However, it is a matter of debate, whether elimination of a powerful cardiovascular risk factor, like a low HS-Omega-3 Index, by a safe and tolerable means, *i.e.*, increased intake of EPA + DHA, should await the advent of the results of a new generation of intervention trials.

### 2.2. Omega-3s and Heart Health—An Australian Perspective—David Colquhoun

In 2003 the American Heart Association recommended 1 g of EPA/DHA for all patients with coronary heart disease and 500 mg per day for asymptomatic individuals.

In 2008 the National Heart Foundation of Australia (NHFA) published its Position Statement. The recommendations were similar to the USA and recommended 1 to 4 g of EPA/DHA per day for lowering triglycerides and to consider omega-3 index.

Since then a number of “neutral” trials which have undermined the evidence supporting the recommendations.

Clinical trials published since 2008: GISSI-HF *n* = 6975, duration 47 months, reduction of mortality: Italian Bypass *n =* 2100, duration 36 months, reduction of mortality: Alpha-Omega *n* = 4837, duration 41 months, no benefit (? Compliance—low dose): OMEGA *n* = 3851, duration 12 months, no benefit (underpowered): SuFol.Om3 *n =* 2501, duration 56 months, no benefit (underpowered): ORIGIN *n* = 12,537, duration 84 months, no benefit (? Compliance): Italian Primary Prevention *n* = 12,513, duration 5 years, no benefit (low major CHD events < 8%—underpowered). 

The European Societies of Cardiology last year withdrew its recommendation of supplemental omega-3 fatty acids in one sentence and referenced only two neutral trials.

The NHFA in 2012 in the Update of Recommendations for Management of Heart Failure Patients recommended 1 g of ethyl ester EPA/DHA as a second line therapy in patients with coronary heart disease on the basis of GISSI-P and GISSI-HF and other trials.

In 2013 the NHFA reconvened its expert committee to review new data. In Australia, NHFA’s Position on omega-3 fatty acids is the most influential amongst clinicians, researchers and the public.

### 2.3. Dietary Fish Oil Reduces Sub-Maximal Heart Rate and Improves Heart Rate Recovery in a Healthy and Fit Population—Michael J. Macartney, Lachlan J. Hingley, Marc Brown, Gregory E. Peoples, Peter L. McLennan

Dietary fish oil incorporates long chain omega-3 polyunsaturated fatty acids (LC*n*-3PUFA); specifically docosahexaenoic acid (DHA) into myocardial membranes and of physiological consequence, heart rate is reduced. However, studies to date have supplemented fish oil at levels unachievable in an average human diet and additionally have focused on cardiovascular based pathologies. Therefore the current study examined whether dietary achievable dosage LC*n*-3PUFA could influence cardiac function in physically fit and healthy humans. Using a double-blind matched design, twenty eight high training male participants were supplemented with (2 × 1 g/day) soy oil (control) or high DHA tuna fish oil (NuMega) (FO), delivering daily: DHA 560 mg and eicosapentaenoic acid (EPA) 140 mg, for eight weeks. Heart rate and blood pressure were recorded during sleep, awake rest, sub-maximal exercise, supramaximal repeat bout exercise (6 × 30 s) and recovery, and a cycling time-trial (5 min). Heart rates recorded during sleep (Control: 50 ± 7, FO: 52 ± 6) or awake rest (Control: 56 ± 10, FO: 59 ± 9) were very low and were not affected by FO. Peak heart rate (Control: 172 ± 11, FO: 174 ± 9) during repeat bout high intensity exercise was also not affected by FO. However, during steady state sub-maximal exercise, total beats over 5 min were reduced in the FO group (−22 ± 6) compared to control (+1 ± 4) (*P* < 0.05). Additionally heart rate recovery after 5 min time-trial cycling was significantly quicker following FO supplementation (−8 ± 2 s) compared to control (Control: −0.4 ± 1 s *P* < 0.05). This study demonstrates that dietary achievable dosage LC*n*-3PUFA reduces heart rate during exercise and improves cardiac recovery responses against a background of high physical fitness in healthy human males.

### 2.4. Omega-3 Index—Beyond Heart Health—Peter R.C. Howe, Alison M. Coates, Jonathan D. Buckley

The Omega-3 Index (O3I: EPA + DHA expressed as percent of total erythrocyte fatty acids) is a novel risk factor for coronary heart disease (Harris WS, *Am. J. Clin. Nutr.* 2008; 87: 1997S) and may also serve as a potential biomarker for other health conditions, including mood and cognitive decline (Milte C *et al.* *Nutr. Rev.* 2009; 67: 573) and body composition (Coates AM *et al*., *Proc. Nutr. Soc. Aust.* 2009; 33: 35). In the latter study we compared omega-3 levels in erythrocytes with measures of adiposity in 135 men and 200 women who had volunteered for clinical trials. There were significant correlations between percent body fat measured by DEXA whole body scans and erythrocyte EPA and DHA levels in both men and women. Body mass index (BMI), a simpler measure of adiposity, was related to erythrocyte DHA in women only. We recently extended this comparison with data from a total of 250 men and 308 women and found in the women that BMI (avg. 33.8 kg/m^2^) correlated strongly with O3I (avg. 5.3%) and DHA (avg. 4.4%) but not with EPA. However, there were no significant correlations in men (BMI and O3I averaged 31.1 kg/m^2^ and 5.0% respectively). These results are consistent with other evidence (e.g., Phang M *et al*., *J. Nutr.* 2013; 143: 457) indicating that omega-3 fatty acids act via gender specific mechanisms. They strengthen previous observations suggesting that low DHA intakes are associated with increased risk of adiposity in women. 

### 2.5. Lipids and Development—Bev Muhlhausler

Fatty acids have long been recognised as essential nutrients for fetal and neonatal development. The omega-3 long chain fatty acids, in particular Docosahexaenoic Acid (DHA: 22:6*n*-3), have been shown to play a particularly important role, and both human and animal studies confirm that severe deficiencies of DHA in the maternal diet are associated with poor health outcomes in the offspring. DHA accumulates rapidly in fetal tissues in the third trimester of gestation, and an adequate supply of this fatty acid is essential for the optimal development of many key organ systems, including the brain and central nervous system. A large number of randomised controlled trials have been carried out to investigate the role of DHA supplementation of the pregnant mother or term/preterm infant on a range of maternal and infant outcomes. This talk will present an overview of the importance of the *n*-3 LCPUFA for fetal and infant development, with a particular focus on more recent evidence suggesting a potential link between increased *n*-3 LCPUFA exposure *in utero* and improved metabolic and cardiovascular outcomes later in childhood.

### 2.6. Neurocognitive Effects of Fish Oil Supplementation in Healthy Adults: Methodological Considerations and Possible Mechanisms of Action—Andrew Pipingas

The abundance of long chain omega-3 fatty acids (LC*n*3) in the brain and retina as well as evidence from epidemiological studies of increased rates of neurologic disease with lower dietary LC*n*3 suggests a role in the maintenance of neurological and cognitive functioning. Intervention studies have shown improvements in cognitive abilities, largely in individuals with a low LC*n*3 status and in more vulnerable groups. In cognitively healthy individuals the results have been mixed, either supporting the premise that LC*n*3 do not improve neurocognitive functioning or suggesting that there may be methodological issues associated with observing an effect, such as dose, duration and sensitivity of the measures used. We have recently completed studies applying brain neuroimaging techniques to investigate effects of different LC*n*3 supplementation on neurocognitive and vascular function in cognitively healthy individuals. Blood fatty acid levels were also measured to explore associations with neurocognitive measures and to investigate inter-subject variability associated with uptake into the blood. The first study that will be discussed was in younger adults focussing on neurocognitive function, applying both brain electrophysiological and functional magnetic resonance (fMRI) imaging techniques. This study was a double-blind crossover design with subjects supplementing their diet with EPA and DHA fish oil (with order of diet randomised) for 1 month. Associations were found at baseline between both cardiovascular and cognitive variables with specific blood fatty acid status including EPA and the AA/EPA ratio; the higher the level of EPA and the lower the level of AA/EPA was associated with better cognitive performance and greater carotid artery blood flow velocity. Supplementation with EPA rich and DHA rich fish oil was associated with differential fMRI activation and brain electrical changes (Bauer I *PLoS One* 2011; 6(12): e28214). The second study was a 4-arm parallel design in 160, 50–70 year old adults investigating 16 week supplementation with different fish oil dosages and in combination with a multivitamin, on blood uptake, neurocognition and cardiovascular function. The large inter-subject variability in the uptake of EPA and DHA into red cells will be the focus of the discussion. Gender contributed to a large proportion of the variability, with females generally showing higher LC*n*3 at endpoint. These findings may partly explain inconsistencies reported in the literature with respect to cognitive endpoints in healthy individuals.

### 2.7. Docosahexaenoic Acid Improves Cognitive Function in Healthy Young Adults—Welma Stonehouse, Cathryn A. Conlon, John Podd, Stephen R. Hill, Anne-Marie Minihane, Crystal Haskell, David Kennedy

Docosahexaenoic acid (DHA), a long chain omega (*n*)-3 fatty acid, is a major component of neuronal cell membranes and affects numerous neuronal and glial cell processess. It is therefore expected that DHA should have cognitive-enhancing effects but robust clinical evidence in younger healthy adults is lacking. Despite it’s critical role in brain function, the capacity to synthesize DHA de novo in humans is limited and it’s consumption through the diet is important to ensure adequate supply for brain function. Individuals following diets low in *n*-3 PUFA may therefore cognitively benefit from DHA supplementation. In addition, gender and apolipoprotein E genotype (*APOE*) impact on cognition and may modulate the response to a DHA supplementation intervention. The study investigated whether a DHA supplement improves cognitive performance in healthy young adults and whether gender and *APOE* modulate the response. Healthy adults (*n* = 176, 18–45 year, non-smoking, low intake of DHA) completed a 6-mo randomized placebo controlled double blind intervention, consuming 1.16 g DHA/day or placebo. Cognitive performance was assessed using a computerised cognitive test battery. For all tests, *z*-scores were calculated and clustered into cognitive domains: episodic and working memory, attention, reaction time (RT) of episodic and working memory and attention and processing speed. ANCOVA was conducted with gender and *APOE* as independent variables. Erythrocyte DHA levels increased significantly in the DHA group compared to the placebo group (mean (95% CI) from 5.28 to 7.91% *vs.* 5.06 to 4.98% respectively, *P* < 0.001) indicating good compliance. RT of episodic and working memory improved with DHA compared to placebo (mean difference (95% CI) −0.18 (−0.33, −0.03) SD, *P* = 0.02; −0.36 (−0.58, −0.14) SD, *P* = 0.002). Gender*treatment interactions occured for episodic memory (*P* = 0.006) and RT of working memory (*P* = 0.03). DHA improved episodic memory in women (0.28 (0.08, 0.48) SD, *P* = 0.006) and RT of working memory in men (−0.60 (−0.95, −0.25) SD, *P* = 0.001) compared to placebo. *APOE* did not affect cognitive function, but there were some indications of *APOE* gender treatment interactions with greater improvements in RTs for working memory and attention in male *APOE4* carriers, but further investigation is needed. In conclusion, DHA supplementation improved memory and RT of memory in healthy young adults whose habitual diet was low in DHA and gender modulated the response to DHA supplementation. Young adults may cognitively benefit from increased consumption of DHA (Stonehouse W *AJCN* 2013; 97: 1134).

### 2.8. The Role of Dietary Fatty Acids in Asthma—Lisa G. Wood

The increase in asthma prevalence in westernised countries in recent decades suggests that environmental factors, such as dietary intake, play a role in the onset and development of the disease. A key feature of westernised diets is an unfavourable fatty acid ratio, with high intake of *n*-6 polyunsaturated and saturated fatty acids and low intake of *n*-3 polyunsaturated fatty acids. This pattern of dietary fat intake provides a pro-inflammatory environment, which may contribute to the development of chronic inflammatory diseases, such as asthma. We have undertaken a series of studies in animal models, aimed at examining how dietary fats modify the inflammatory response to common asthma triggers. In a model of respiratory virus-infected cultured epithelial cells, we demonstrated that *n*-3PUFA supplementation resulted in a reduction in IL-6 and IP-10 release after rhinovirus infection (Saedisomeolia, *BJN*, 2009; 101: 533–540). In an allergic mouse model, we demonstrated that *n*-3 PUFA supplementation reduced eosinophilic infiltrates in BAL fluid and lung tissue, reduced mucus hypersecretion and reduced airway hyperresponsiveness in response to an allergic trigger (Wood, *Clin. Exp. All* 2010; 40: 1785–1793). We have also investigated the role of saturated fat in the airways of adults with asthma. Non-obese subjects with asthma were randomised to consume a high fat (48%) or low fat (15%) meal. The high fat meal was associated with activation of innate immune responses in the airways, including increased airway neutrophilia and TLR4 gene expression (Wood LG, *JACI* 2011; 127: 1133–1140). We conclude that reduced saturated fat intake and increased *n*-3PUFA intake may attenuate airway inflammation in asthma, thereby providing a useful addition to current asthma management strategies.

### 2.9. Fish Oil in Recent Onset Rheumatoid Arthritis: A Randomized, Double-Blind, Controlled Trial—Susanna Proudman, Les Cleland, Llew Spargo, Cindy Hall, Leah McWilliams, Anita Lee, Michael James

Traditional randomized controlled trials (RCT) of fish oil (FO) in rheumatoid arthritis (RA) were problematic in that patients had longstanding disease (average duration approx 10 years) and the need to change dosing of the disease-modifying anti-rheumatic drugs (DMARDs) was usually a withdrawal criterion. We have addressed these shortcomings in a double-blind RCT of FO in RA by using patients with recent onset disease and using an algorithm for drug dosing that takes into account disease activity as well as drug toxicity/intolerance.

140 Patients with active RA of <12 months’ duration and who were DMARD-naïve were randomized 2:1 to fish oil or control. The fish oil dose provided 5.5 g/day of the omega-3 fats, EPA and DHA. The control was a monounsaturated oil containing a low dose of fish oil providing 0.4 g/day EPA + DHA for masking. All participants received the DMARDs methotrexate, sulphasalazine and hydroxychloroquine (“Triple Therapy”) with doses adjusted according to the algorithm based on disease activity and toxicity. The primary outcome measure was the failure of triple DMARD therapy in the first 12 months.

Failure of triple DMARD therapy was significantly less in the fish oil group compared with the control group (Hazard Ratio = 0.28 (95% CI 0.12–0.63; *P* = 0.002). Also, the rate of remission was significantly greater in the fish oil group compared with the control group (Hazard Ratio = 2.17 (95% CI 1.07–4.42; *P* = 0.03).

This novel RCT design has revealed beneficial effects of anti-inflammatory doses of fish oil used adjunctively with modern RA therapy.

### 2.10. Omega-3 Market Update—Adam Ismail

The market for omega-3 oils has grown considerably over the past four decades, driven by science and the resulting advances in consumer awareness. Despite this growth, the vast majority of the earth’s population does not consume sufficient levels of omega-3s. The industry can continue to grow by addressing the needs of nonusers, but these needs vary from country-to-country. However, if demand continues to increase, the industry will also need to ensure that it can supply sufficient quantities of EPA and DHA omega-3s. The market has traditionally been dominated by anchovy-sourced oils, but growth in demand for these oils will start to reach limits of what these fisheries can sustainably supply. This scenario is triggering innovation and increased diversity of supply, with dozens of plant, microbial and new marine sources having been launched in the past decade. However, the capacity of any of these sources to contribute meaningfully to human nutrition needs is small, so the manufacturers leveraging these sources are differentiating based on the unique characteristics of the oil produced. This presentation will explore the dual challenges of needing to increase demand and simultaneously increase supply of a natural resource.

### 2.11. Aquaculture and LC Omega-3—Giovanni M. Turchini

LC Omega-3 are beneficial to human health and their consumption is advocated globally. However, the only readily available source of LC Omega-3 is seafood, and in the last two decades, the global landing of wild seafood has been stagnating. On the other hand, the rapid expansion of aquaculture, outpacing global population growth, has been responsible for increased global seafood availability (total and per-capita). However, aquaculture is a highly diverse industry, and whilst some sectors positively impact on the global seafood availability, other sectors, which rely on aquafeed formulated with the inclusion of wild caught derived marine raw materials, can have a negative impact on the global fish and seafood supply, and even more so for the actual LC-omega-3 availability. The background economic situation is very simple, and a perfect example of the first basic law of supply and demand: the demand for LC-Omega-3 is increasing (aquaculture, human consumption, pharmaceutical and animal nutrition) and its supply remains unchanged (wild fishery). Thus the world is facing a shortage of LC-Omega-3, leading to a constantly higher equilibrium price. Whilst detailed information on the past, present and predicted fisheries landings and aquaculture production is available and intense scientific debates and focused research effort are addressing this issue on a fish input/output (weight/weight) balance, little is known specifically for the LC Omega-3 input/output balance, and in general LC Omega-3 availability and sustainable use. This paper aims at briefly reviewing current knowledge on this topic, and attempts predictions of possible future outcomes, constrains and advancements. 

### 2.12. Readily Available Sources of Long-chain Omega-3 Oils: Is Farmed Australian Seafood a Better Source of the Good Oil than Wild-Caught Seafood?—Peter D. Nichols, James P. Petrie, Surinder P. Singh

Nutritionists and medical authorities encourage seafood consumption as the oils contain nutritionally important omega-3 long-chain polyunsaturated fatty acids (LC-PUFA, also termed LC omega-3 oils). Wild-caught seafood is promoted as an ideal source of the two important LC omega-3—EPA and DHA. The human body manufactures only small amounts of these LC-PUFA, so we need them from our diet. Two volumes of the FRDC-funded Guide “Seafood the Good Food” detailed oil and PUFA composition of Australian seafood, with species examined largely from the wild. The Guides also provided an indication to LC omega-3 cotent in farmed species. Concern has been expressed in recent times that cultured (farmed) fish contain lower LC omega-3 content than wild-harvested seafood. We examined the current state of play with cultured Australian seafood. Under current feeding practices, farmed Australian finfish (Atlantic salmon, barramundi) generally have higher oil and LC omega-3 content than either the same or other species from the wild, and remain one of the best ways to achieve substantial dietary intake of the LC omega-3 oils. Notwithstanding, the content of the LC omega-3 oils has decreased over the past decade in farmed Australian seafood. This has largely resulted from replacement of dietary fish oil with chicken fat. For Atlantic salmon, LC omega-3 content has decreased by 50% or more since 2002, and the 3/6 ratio has decreased from >5 to <1. The exciting prospects for the development of oilseeds containing LC omega-3 oils can in the future allow these health-benefitting oils to be maximized in farmed Australian seafood, with such advances capable of assisting with preventative health care, fisheries management, aquaculture nutrition, an innovative feed and food industry, and ultimately towards improved consumer health. As many Australian consumers increasingly seek their LC omega-3 from supplements, an overview of selected supplement products also will be presented.

### 2.13. Integrated Processing for the Recovery of High Value Lipids from Biomass—Owen Catchpole, Stephen Tallon, Jagan Billikanti, Peter Dyer, Andrew MacKenzie

This presentation reviews our recent work on the integration of upstream and downstream processing with both near-critical and conventional solvent extraction to obtain specific high value lipids in high concentrations. Upstream processes include fermentation, enzyme pre-treatments, particle size reduction, slurry formation and drying. Downstream processes include purification and fractionation, chemical and enzymatic lipid conversion, and lipid concentration. The aim of the work was to optimise the whole suite of technologies with regard to processing costs, product yield and product quality. The integration of upstream and downstream processing with extraction is demonstrated in detail for the production of EPA ethyl esters in high concentration from marine microalgae biomass. Other examples of extraction and concentration of specific lipids and carotenoids will also be presented. The production of very high purity EPA represented a considerable challenge, due to the complexity of the lipids in the biomass, the nature of the biomass, and the degree to which the EPA needed to be purified. We were able to achieve a >95% EPA product through a combination of upstream process technologies, near-critical extraction using dimethylether or conventional extraction using isopropyl alcohol, chemical conversion of lipids to ethyl esters followed by supercritical chromatography.

### 2.14. Novel Resolvin and Protectin Analogues from EPA and DHA—A More Bioactive Source of Omega-3—Polly Dobson, Colin J. Barrow, Jaroslav A. Kralovec and Jacqui L. Adcock

The health benefits of omega-3 fatty acids are widely recognized although their mechanisms of action are still not fully understood. In the last fifteen years, endogenous lipid mediators involved in the resolution of inflammation were discovered and termed resolvins and protectins (Serhan, C.N., *J. Exp. Med.* 2002; 196: 1025). These compounds, derived from DHA and EPA, have helped to explain the myriad health benefits associated with omega-3s. Resolvins and protectins are transiently expressed hydroxylated compounds synthesized from endogenous DHA and EPA by up-regulated oxygenase enzymes. These lipid mediators have been shown to be orders of magnitude more biologically active than their precursors in both resolving inflammation and protecting tissue from the associated damage. The potential of synthesizing novel and known resolvin and protectin analogues for supplementation, is an attractive alternative to the currently available concentrated fish and krill oils. In this work we have developed a simple and effective method for controlling the progression of resolvin and protectin analogue synthesis utilizing an isolated 15-lipoxygenase enzyme (15-sLOX-1) from soybean (*Glycine max*). We have investigated the effect of experimental conditions on the catalytic activity of 15-sLOX-1, with pH and enzyme concentration found to have a significant effect on product formation, giving rise to the development of two methods for the controlled synthesis of mono and dihydroxy compounds from five biologically significant PUFAs (Dobson, E.P., *J. Lipid Res.* 2013; 54: 1439–1447). The described methods could be further developed in the synthesis of these mediators from hydrolyzed fish and krill oils, which are high in DHA, EPA and DPA using crude lipoxygenase sources. This offers an economical and “green” method for large scale production of anti-inflammatory mediators from natural products.

### 2.15. Composition of Antarctic Krill Oil and Methods for its Harvesting, Production and Qualitative and Quantitative Analysis—Nils Hoem

Antarctic krill is an excellent source of omega-3 lipids. The omega-3 fatty acids in krill oil are mainly present in phospholipid form. This makes krill oil distinct from other sources of omega-3 fatty acids such as fish oil, where the omega-3 fatty acids a present in triglyceride or ethyl ester form. To ensure rapid processing in a food certified processing plant of this fragile and auto-digesting raw material, Aker BioMarine utilizes a proprietary harvesting technology that brings the krill alive on board the factory vessel. Most importantly, the technology prevents by-catch, and our krill fishery is now certified sustainable by the Marine Stewardship Council. Krill can therefore be a rich and sustainable source of omega-3 fatty acids important for human health. Processing as well as chemical analysis of krill oil requires methods that are distinctly different from common practice in the fish oil industry. There is now evidence that omega-3 fatty acids in form of phospholipids are more bio-efficient than the corresponding omega-3 fatty acids in the form of triglycerides and ethyl esters.

### 2.16. Novel Formulations and Process for Development of Microencapsulated Krill Oil Emulsion and Powder—Luz Sanguansri, Zhiping Shen, Sukhdeep Bhail, LiJiang Cheng, DanYang Ying and Mary Ann Augustin

The well recognized health benefits of omega-3 fatty acids in our diets continue to create demand for development of stable omega-3 ingredients to facilitate the delivery of omega-3 fatty acids through foods or supplements. Microencapsulation protects and stabilise omega-3 fatty acids from oxidation, masks undesirable taste and aroma and provides a convenient format in powder form. Selection of encapsulant materials and processes used for microencapsulation of omega-3 oils is influenced by the type and source of the oil being encapsulated. We previously reported the oxidative stability of microencapsulated fish oil powders within matrices of heated mixtures of protein and carbohydrate, and its ability to mask undesirable flavour and odour when incorporated in food. In this presentation we will discuss our more recent progress on the development of microencapsulated Krill oil rich in phospholipids, and present results on its stability, and performance during extrusion and compression.

### 2.17. Production of the Omega-3 Eicosapentaenoic Acid through Heterotrophic Fermentation—Hywel Griffiths, Kathrine Allikian, Mark Dines, Moreland Gibbs

Average global fish oil production has been static for the last 30 years, but increasing demand from aquaculture and, more recently, human nutrition means that supply is becoming constrained. Pharmaceutical uses of high purity omega-3s, especially eicosapentaenoic acid (EPA), are also growing, and adding to the demand.

Microalgae, as the primary source of marine-derived omega-3 fatty acids, offer tremendous opportunity to bypass the limitations of fish oil, and indeed some characteristics of algal sources might be preferred for medical uses over fish based products. Production of microalgae, however, presents considerable challenges due to their unique growth requirements and relative novelty in the bioprocessing industry. Photonz is a New Zealand company that has developed a process for production of high-purity EPA from an algal source, and with commercial-scale production in progress, we are positioned to be a supplier of high-purity, algal-derived EPA for the clinical markets.

### 2.18. Engineered Oil Seed Crops with Fish Oil like DHA Levels—Surinder Singh, Peter Nichols and James Petrie

Omega-3 long chain (≥C_20_) polyunsaturated fatty acid (ω3 LC-PUFA) have critical roles in human health and development with studies indicating that deficiencies in these fatty acids can increase the risk or severity of cardiovascular and inflammatory diseases in particular. These fatty acids are currently predominantly sourced from fish and algal oils. In order to meet the increasing demand for these oils there is an urgent need for an alternative and sustainable source of EPA and DHA. This talk will discuss recent progress in the production of the ω3 LC-PUFA DHA, in plant seeds. Groups have reported good progress in engineering the C_20_ EPA with seed fatty acid levels similar to that observed in bulk fish oil (~18%) although undesirable ω6 PUFA levels have also remained high. The conversion of EPA to the particularly important C_22_ DHA, however, has been problematic with many attempts resulting in the accumulation of EPA/DPA but only a few percent of DHA. I will describe the production of fish-oil like levels of the C_22_ fatty acid DHA in seed oils of model and oilseed crop species with high omega-3/omega-6 ratios. Importantly, these results were achieved using a single multi-gene construct which potentially will allow for a simpler pathway for deregulation and breeding. We consider this to be a breakthrough in the development of sustainable alternative sources of DHA.

### 2.19. Dietary Modelling of Omega-3 Enriched Foods on Omega-3 Intake of Australian Children—Barbara J. Meyer, Setyaningrum Rahmawaty, Philippa Lyons-Wall, Karen Charlton

In countries with traditionally low fish consumption such as Australia, foods enriched with omega-3 long chain polyunsaturated fatty acids (*n*-3 LCPUFA) may play an important role in meeting *n*-3 LCPUFA intakes for optimal health. The aim of the present study was to assess the effect of replacement of bread, egg, milk and yogurt with *n*-3 enriched alternatives on total *n*-3 LCPUFA intake in Australian children’s diets. Dietary modelling was undertaken using survey data from a nationally representative sample of 4487 children (2249 boys, 2238 girls) aged 2–16 years. Fifteen models were constructed in which reported consumption of bread, egg, milk and yogurt was replaced with *n*-3 enriched alternatives that are commercially available. Mean ± SD and median (IQR) intakes of *n*-3 LCPUFA gradually increased from 79.2 ± 173.1 to 104.6 ± 178.3 mg/day and 28.9 (10.9–21.8) to 51.4 (25.7–109), respectively, after the modelling (*p* = 0.001 for each model). Median (IQR) intake of total LCPUFA’s in non-fish eaters and fish eaters were 20.8 (8.1–42.7) and 150 (76–308) mg/day and these increased by 19.0 mg (91%) to 39.8 (22.5–73.6) and 33 mg (22%) to 183 (97.7–332) mg/day respectively after replacement of all four items. The proportion of children that met the adequate intake (AI) and the suggested dietary target (SDT) for *n*-3 LCPUFA increased by 15.2% and 0.4%, respectively. Replacement of four commonly consumed food items with *n*-3 enriched alternatives resulted in doubling the *n*-3 LCPUFA intakes in Australian children, without changing to their current food habits.

### 2.20. Food and Fuel Forever—Julian Cribb

Feeding 10 billion people sustainably through the latter half of the 21st century will present the greatest challenge humanity has ever faced. With food and energy demand set to double by 2060, critical scarcities and instabilities are emerging in almost all the key resources required to satisfy it, oil especially. There is a cluster of “ticking timebombs” with far-reaching strategic significance that challenge us to rethink food itself, how we produce it, and how to create diets, food and energy for the future in ways that are safe, healthy, use fewer resources and tread less heavily on the planet. In this talk I explore ways that Australian can guarantee its production of food and transport fuels into the future in spite of changes in climate and geopolitics.

### 2.21. Australian Oilseed Production—Supplying the World’s Hungry Mouths and Thirsty Engines—Nick Goddard

Australian arguably produces some of the best quality canola in the world—bred specifically to be highly nutritious—low in saturated fat and with good levels of essential fatty acids—in particular, omega-3. With Australia being the number 2 exporter of canola in a world where cardio vascular disease is the number one killer in developed countries, and rapidly climbing to number one in developing countries, Australian canola oil has a strong role to play in improving global health.

There is certain irony, therefore, when the majority of Australian canola is used to feed, not men and women, but Volvos and Mercedes. For the past few years, with record domestic production, millions of tonnes of Australian canola has been shipped to Europe to meet the mandated biodiesel requirements of EU states.

The moral dilemma of food *versus* fuel has rarely entered the minds of exporters or growers as the European buyers pay premium for Australian canola for biodiesel.

Is this the future of Australian canola production—for growers across the country to take on the price and production risk to meet the distorted market demand for biodiesel in Europe? If this is the future for Australian canola, what signals does this send to breeders? Or are the winds of change beginning to blow—and will we see more fundamental market drivers determining the future of the Australian canola crop.

This keynote address will outline the opportunities for Australian canola in the context of a dynamic and fast changing global market.

### 2.22. Oil for Food or Fuel in New Zealand and the Myth of Marginal Soils—Jeff I. McCormick, Bruce Smallfield, Vonny Fasi and Peter Tait

The use of arable soils for the production of oilseed crops for biodiesel has often been criticized due to concerns of reducing food produced for human consumption. In contrast, the use of marginal soils to produce oilseed has often been promoted as a more socially acceptable method. Often these soils are perceived by the general public as “spare” land with no alternate purpose. In New Zealand farming systems, many of these soils in actual fact are highly utilised by the pastoral industry to produce high value animal protein, both meat and milk. Changing the land use on these soils would require large changes in farming systems and lead to increased risk to individual farmers. Conversely utilising arable soils with high yield potential would limit the land area required for the production of biodiesel with agronomic benefits for the other crops in the rotation. The regional scale production of biodiesel for transport and farm use could lead to price certainty for fuel, a large input cost on farms. We will examine this concept in relation to the production of Oilseed rape (*Brassica napus*) and Camelina (*Camelina sativa*) on arable and marginal soils. We demonstrate in this paper that the production of biodiesel on a regional scale from arable cropping land could lead to more resilient farms without a decrease in global food security.

### 2.23. A Step-Change in Biomass Oil Accumulation: Oilseed TAG Yields from Leaves—James R. Petrie, Thomas Vanhercke, Surinder Singh

Demand for plant oils will increase rapidly as the population grows in the coming decades. Limitations on arable land and other inputs mean it may be difficult to meet this additional demand with current oilseed-based production systems. In response, there has been significant investment in the production of high biomass plants with elevated triacylglycerol (TAG) content for both food and oleochemical (fuel and feedstock) applications. We report the production of Nicotiana tabacum events which have been genetically modified to produce in excess of 10% TAG in their aerial biomass. This was achieved by combining a variety of oil increase technologies in a single, coordinated approach to effectively overcome the “Push” (fatty acid synthesis), “Pull” (TAG assembly) and “Accumulation” (TAG storage) limitations in plant cells. We describe surprising synergistic effects between technologies as well as implications on efforts to achieve TAG secretion in plants. We also describe how the TAG profile has shifted significantly away from polyunsaturated fatty acids toward saturated and monounsaturated fatty acids and describe broader changes to the lipidome. We consider this to be a breakthrough toward the production of a new and viable plant oil production platform that does not necessarily compete with existing farmland.

### 2.24. High Purity Oleic Acid from GM Safflower Seed—Craig Wood, Qing Liu, Matt Taylor, Shoko Okada, Xue-Rong Zhou, Allan Green, Surinder Singh

Oleic acid is found in all plant-based food oils, but does not naturally reach the very high levels of purity that are needed for expanding its use as a petrochemical replacement in production of polymers, lubricants and cosmetics. We have addressed this limitation by using genetic engineering to produce safflower seed oil with over 94% oleic acid content, with reduced levels of palmitic acid and minimal linoleic acid. In Australia, safflower is considered an excellent crop platform for industrial oil products because it is only a minor food crop and has favourable reproductive biology for transgenic trait containment. Deep sequencing of safflower DNA and RNA combined with biochemical characterisation efforts were used to determine the genes most likely to contribute to linoleic acid production in safflower seed. Surprisingly, safflower has an expanded family of FAD2 genes, comprising at least 11 members, with only some functioning as oleic acid desaturases in developing embryos. Safflower also possesses at least three FATB genes, with two members expressed in developing embryos. Long fragments of the seed-expressed FAD2 and FATB genes were used to design an RNAi silencing construct, driven by a seed-specific promoter, to ablate FAD2 and FATB activities in safflower seeds. Elite events generated safflower seeds oils with near-pure levels of oleic acid, whilst lipid profiles in other vegetative tissues remained unaffected. These materials are currently being evaluated in field trials to assess their agronomic performance relative to non-GM check varieties and for the generation of oil samples for functional testing.

### 2.25. Molecular Basis for Differential Elongation of Omega-3 Docosapentaenoic Acid by the Rat Elongases Elovl5 and Elovl2—Melissa K. Gregory, Leslie G. Cleland, Michael J. James

Metabolism of α-linolenic acid (18:3*n*-3; ALA) to eicosapentaenoic acid (20:5*n*-3; EPA) and docosahexaenoic acid (22:6*n*-3; DHA), requires progressive desaturation and elongation. Functional characterisation of the rat elongases, Elovl5 and Elovl2, have identified that Elovl2 is crucial for omega-3 DHA synthesis (Gregory M. *PLoS One* 2011; 6(12): e29662). Whilst the substrate specificities of the rat elongases had some overlap, only Elovl2 can convert the C_22_ omega-3 PUFA docosapentaenoic acid (DPA) to 24:5*n*-3, which is the penultimate precursor of DHA. We examined the molecular reasons for the differences between Elovl5 and Elovl2 in their ability to elongate DPA to 24:5*n*-3. Using a yeast expression system we examined a series of Elovl2/Elovl5 chimeras and point mutations to identify Elovl2 residues which are responsible for DPA substrate specificity. The results indicate that the cysteine at position 217 in Elovl2 and a tryptophan at the equivalent position in Elovl5 explain their differing abilities to elongate DPA to 24:5*n*-3. Further studies confirmed that Elovl2 C217 is a critical residue for elongation of DPA at the level observed in the native protein. Understanding the ability of elongases to synthesise 24:5*n*-3 may provide a basis for using sequence data to predict their ability to ultimately support DHA synthesis.

### 2.26. Comparative Responses to Very Low Fish Oil Supplementation in Heart and Skeletal Muscle: Effect of Dose, Duration and Fibre Type—Peter L. McLennan, Renee Henry, Gregory E. Peoples

Cellular membrane incorporation of polyunsaturated fatty acids (PUFA) influences cellular signaling and physiological function. It depends upon diet and also shows tissue specificity. Excitable cells such as heart and skeletal muscle readily incorporate long chain (LC) *n*-3PUFA docosahexaenoic acid (DHA) from high dietary fish oil intakes. Recent evidence shows myocardial DHA incorporation increases even in response to very low dietary availability of LC*n*-3PUFA. We tested the hypothesis that skeletal muscle is similarly sensitive to low-dose fish oil feeding. Male Wistar rats were fed for 15 w on 10% fat diets containing: olive oil alone (control); 0.31% (lowFO); or 1.25% moderate (modFO) NuMega, high-DHA Tuna Fish Oil in olive oil, delivering linoleic acid (LA) as 1.92, 1.87, or 1.72%en and DHA as 0, 0.20 and 0.83%en at dietary *n*-6/*n*-3PUFA ratios of 16.6, 5.1 and 1.6 respectively. The DHA relative phospholipid fatty acid concentration was greater in control gastrocnemius muscle (9.3 ± 0.7%) than in soleus muscle (5.1 ± 0.2%) or myocardium (6.6 ± 0.3%). Feeding lowFO or modFO markedly increased DHA (gastrocnemius: 19.9 ± 0.4%, 24.3 ± 1.0%; soleus: 14.3 ± 0.7%, 18.0 ± 1.4%; myocardium: 16.6 ± 0.4%, 20.4 ± 0.7%). Proportions of *n*-6PUFA arachidonic acid declined with both FO doses but linoleic acid was significantly reduced only with modFO. All muscle tissues were highly responsive to very small intakes of LC*n*-3PUFA in the achievable human dietary range. Soleus and myocardium showed similar fatty acid incorporation patterns, different to gastrocnemius. This may relate to their slow twitch, oxidative, fatigue-resistant characteristics compared to the fast, glycolytic, fatigable metabolic physiology phenotype of gastrocnemius and may be expressed in different physiological responses.

### 2.27. Association between Whole Blood Omega-3 Fatty Acids and Type 2 Diabetes—Amani Alhazmi, Manohar L. Garg, Elizabeth Stojanovski, Mark McEvoy

Limited data exist on the use of objective biomarkers of fatty acids for assessing the association between dietary intake of omega-3 polyunsaturated fatty acids (*n*-3PUFA) and type 2 diabetes risk. The aim of this study was to investigate the association between whole blood *n*-3PUFA concentrations and incident type 2 diabetes in older adults. A nested case-control study of 37 cases of type 2 diabetes and 150 controls was conducted. Data were analyzed from a cohort of older adult aged 55–85 years participating in the Hunter Community Study. Whole blood *n*-3PUFA was measured using gas chromatography and incident diabetes was ascertained by self-reported questionnaire at the baseline of the study. Blood concentration of *n*-3PUFA (ALA, EPA, and DHA) was higher in people with type 2 diabetes compared to the non-diabetic (control) participants. The multiple adjusted odd ratios of type 2 diabetes were (OR = 1.08, 95% CI: 1.02–1.15, *P* = 0.02) for the ALA; (OR = 1.05, 95% CI: 1.02–1.08, *P* = 0.0004) for EPA; and (OR = 1.03, 95% CI: 1.02–1.05, *P* < 0.0001) for DHA. These results suggest that higher whole blood concentrations of *n*-3PUFA (ALA, EPA, and DHA) were associated with increased risk of diabetes in older adults. 

### 2.28. Acute Effects of Feeding Fructose, Glucose and Sucrose on Blood Lipid Levels and Systemic Inflammation—Faizan Jameel, Melinda Phang, Lisa G. Wood, Manohar L. Garg

Recent studies have demonstrated a relationship between fructose consumption and risk of developing metabolic syndrome. Mechanisms by which dietary fructose mediates metabolic changes are poorly understood. This study compared the effects of fructose, glucose and sucrose consumption on post-prandial lipemia and inflammatory markers. The trial design was a randomized controlled, cross-over intervention involving equal numbers of healthy male and female adults (*n* = 14). After an overnight fast, participants were given a drink containing 50 g of either fructose or glucose or sucrose dissolved in water. Blood samples were collected at baseline, 0.5, 1 and 2 h post intervention for the analysis of blood lipids, glucose, insulin and high sensitivity C-reactive protein (hs-CRP). Glucose and sucrose supplementation initially resulted in a significant increase in glucose and insulin levels compared to fructose supplementation and returned to near baseline values within 2 h. The change in plasma total cholesterol, LDL and HDL-cholesterol (measured as area under curve, AUC) was significantly higher when participants consumed fructose compared with glucose or sucrose (*P* < 0.05). Plasma triglyceride (measured as AUC) levels however remained unchanged regardless of the dietary intervention. The change in hs-CRP (measured as AUC) was also significantly higher in subjects consuming fructose compared with those consuming glucose (*P* < 0.05), but not sucrose (*P* = 0.07). A single dose of fructose increases post-prandial lipemia and systemic inflammation in comparison to glucose and sucrose. This study was funded by Jenny Thomas Trust funds via Hunter Medical Research Institute (HMRI).

### 2.29. Omega-3 Polyunsaturated Fatty Acids Alleviate Dietary Saturated Fat-Induced Postprandial Rise in Blood Lipid Levels—Cintia B. Dias, Lisa G. Wood, Melinda Phang, Manohar L. Garg

Saturated fatty acids (SFA) have been associated with elevated blood lipid levels, although animal studies have demonstrated that dietary SFA raise blood lipid levels only when the diet is deficient in omega-3 polyunsaturated fatty acids (*n*-3PUFA). Therefore, we investigated the postprandial effects of *n*-3PUFA supplementation on plasma lipid profile when the background diet was high either in SFA or in omega-6 polyunsaturated fatty acids (*n*-6PUFA). This was a randomised controlled, cross-over, dietary intervention trial involving 17 healthy females aged 18 to 65 years. Blood was collected after an overnight fast, then subjects consumed a single meal consisting of 3 capsules of *n*-3PUFA (1.8 g), 200 mL water and 150 g mashed potato mixed with either 38 g butter (high SFA) or 32 g sunflower oil (high *n*-6PUFA). Blood samples were then collected at 1, 3, 4 and 6 h post meal consumption. After at least one week washout, the same procedure was repeated, following consumption of the alternate meal. Blood lipid profile (cholesterol, LDL-cholesterol, HDL-cholesterol and triglycerides) was measured at each time point. Change in plasma triglycerides (measured as area under the curve) was significantly higher when participants consumed *n*-6PUFA plus *n*-3PUFA, compared with the SFA plus *n*-3PUFA meal (*P* = 0.0309). In addition, no significant difference was observed in cholesterol levels. Thus, *n*-3PUFA supplementation appears to be more effective in controlling post-prandial levels of plasma triglycerides in females, when the background diet contains SFA rather than *n*-6PUFA.

### 2.30. Dietary Fish Oil Protects Skeletal Muscle from Hypoxic Stress during about of Contractile Fatigue in the Rat *in vivo* Hindlimb—Gregory E. Peoples, Peter L. Mclennan

Under normoxic conditions, dietary fish oil modifies skeletal muscle membrane fatty acid composition and improves oxygen (O_2_) efficiency enabling sustained contractile force. Oxygen efficiency is a key determinant of force production in mammalian skeletal muscle during hypoxic stress. Therefore we examined the protective effects of fish oil diet on skeletal muscle fatigue under the stress of hypoxia the rat *in vivo* autologous perfused hindlimb. Male Wistar rats were fed for eight weeks on a diet rich in saturated fat (SF), long chain (LC) *n*-6 polyunsaturated fatty acids (PUFA), or LC *n*-3 PUFA docosahexaenoic acid (DHA) from fish oil. In anaesthetised, mechanically ventilated rats (normoxia 21% O_2_ and hypoxia 14% O_2_) with the hindlimb perfused with arterial blood at a constant flow, the gastrocnemius-plantaris-soleus muscle bundle was stimulated via sciatic nerve (2 Hz, 6–12 V, 0.05 ms). Independent of diet, hypoxic conditions caused a reduction in PaO_2_ to <70 mmHg (*P* < 0.01 v normoxia) and attenuated peak twitch tension (normoxia: 82 ± 8; hypoxia 41 ± 2 g/g tissue w.w). However under hypoxic stress, rats fed the LC *n*-3 PUFA diet were protected against the extent of tension decline whereby they sustained higher maximum twitch tension compared to the SF and *n*-6 PUFA groups (*P* < 0.05) and completed more contractions before decline to 50% of maximum twitch tension (SF; 546 ± 58, *n*-6PUFA; 522 ± 58, *n*-3 PUFA; 792 ± 96 seconds; *P* < 0.05). These results further support the role of DHA in skeletal muscle membranes under stressful conditions and this is expressed as an increased sustained force production and prolonged time to fatigue.

### 2.31. Lipase-Catalysed Synthesis of Omega-3 Fatty Acid Concentrate in Acylglycerol from Fish Oil—Taiwo O. Akanbi, Jacqui L. Adcock, Colin J. Barrow

Clinical benefits of concentrates of omega-3 fatty acids, such as eicosapentaenoic acid (EPA) and docosahexaenoic acid (DHA), in the treatment and prevention of health disorders such as cardiovascular, Alzheimer’s and Parkinson’s diseases have made them the subject of intensive research. Current techniques for the production of omega-3 concentrates are expensive and environmentally unfriendly involving fractional distillation and urea complexation techniques. In pursuit of cheaper, milder and greener techniques for concentrating omega-3 fats, we investigated the use of a commercial lipase for concentrating EPA and DHA via fish oil hydrolysis. Monitoring percent hydrolysis using capillary chromatography with flame ionization detector (Iatroscan) and omega-3 concentration using gas chromatography (GC) indicated that during hydrolysis DHA primarily remained on the glycerol backbone, while EPA was progressively removed. ^13^C nuclear magnetic resonance (NMR) data showed a clear increase in DHA at all positions (*sn*-1,3 and *sn*-2) which resulted in a 2-fold increase in its concentration (Akanbi *et al.*, *Food Chem*. 2013; 138: 615–620).

### 2.32. Characterisation of Lipase Fatty Acid Selectivity Using Novel Omega-3 pNP-acyl Esters—Tim D. Nalder, Susan Marshall, Frederick M. Pfeffer, Colin J. Barrow

Lipases have applications for the industrial processing of lipids, including concentrationand/or modification of omega-3 fatty acids (FA), the main source of which is fish oil. A range of para-nitrophenol (*p*NP) acyl esters were synthesised as a means to rapidly screen lipases for FA selectivity using spectrophotometric detection. The chosen esters were based primarily on the most abundant fatty acids present in anchovy and tuna oils. *p*NP derivatives of 16:1 *n*-7, 18:1 *n*-9 (OA), 18:2 *n*-6 (LA), 18:3 *n*-3 (ALA), 20:5 *n*-3 (EPA) and 22:6 *n*-3 (DHA) were synthesised. Storage stability of these *p*NP derivatives was shown to be at least 6 months and all *p*NP derivatives, including those of EPA and DHA, were shown to be stable throughout the conditions of the assay. We applied the new assay substrates for the determination of fatty acid selectivity of five widely utilised lipases. Results showed that the lipase from *Candida rugosa* was the most selective in terms of omega-3 specificity, preferentially hydrolysing all other medium-long chain substrates. Lipases from *Rhizomucor miehei* and *Thermomyces lanuginosa* also showed selectivity, with a significant preference for saturated fatty acids. *Candida antarctica* lipase B and *Aspergillus niger* lipase were the least selective.

### 2.33. Tailored Liposomes for Optimised Biomaterials Delivery—Rahau S. Shirazi

Liposomes have been identified for nano-scale delivery of biomaterials and are receiving worldwide interest. Optimum transfer in target media is directly influenced by the structure of liposome building blocks, namely lipids. Thus, the design and assessment of liposomal characteristics, either as simple liposomes or in complexes with biomaterials, are probed using varied techniques (e.g., Environmental Scanning Electron Microscopy). The development of liposomes containing novel natural complex lipids with unique features can enhance liposomal bioactivity. Hence, strategic modifications in the development of liposomes employ a wide variety of novel natural complex as well as synthetic lipids. The chemical structure of each synthetic lipid compartment can correspondingly influence the stability, safety and efficiency of developed liposomes, also influencing the liposome’s ability to encapsulate, protect and deliver biomaterials. A series of cationic-stealth liposomes have been prepared using varied PEG-PE chain lengths, cationic and neutral lipids (*i.e.*, DC-Chol, DOTAP, CHEMS, DOPE, DOPC and DODAP) along with the relevant non-PEGylated liposomes. Characterisation studies of these systematically prepared liposomes have led to the identification of stable liposomes with optimised encapsulation ability for biomaterials. Incorporation of novel natural complex lipids as well as stimuli-responsive lipids into such systems has led to the production of tailored liposomes with distinctive characteristics. Liposomes developed using these strategic modifications can perform as super nano-vectors for microencapsulation in the cosmetics and drug industries.

### 2.34. Development of a Frying Oil Stability Model—Prakash Adhikari, Paul Smith, Sean Smith, Andreas Menzel

The life time of frying oil is determined by its stability against oxidation. National laws in many countries have defined quality parameters which determine whether oil can still be used or needs to be replaced. For example, European countries have established the level of 24%–27% polar value as the rejection point for the heated oils (Paul S. *Crit. Rev. Food Sci.* 1997; 37: 635). At the same time there is a significant push to more healthy oils, which contain higher proportions of unsaturated fatty acids. This causes a risk of reduced oxidative stability of these healthier frying oils. In order to being able to predict relative oxidative stability of different frying oil blends, we have developed a statistical model to serve as a tool that enables a realistic estimation of the relative oxidative stability of a different oil blends prior to intensive frying tests. The statistical model was built using an accelerated oil oxidation reactor, following fundamental oil oxidation parameters, including peroxide value and anisidine value. The analytical results were all fit into a statistical model by determining the kinetics parameters for many different oxidation equations, in order to achieve a tool enabling realistic predictions.

### 2.35. Simulation of Industrial Frying of Potato Chips Using a Pilot Plant Continuous Fryer: Physico-Chemical Changes in Palm Olein Binary Blends under Continuous Frying Conditions—Azmil Haizam Ahmad Tarmizi, Razali Ismail

Binary blends of palm olein with sunflower oil (SFO), canola oil and cottonseed oil (CSO), respectively were formulated to assess their performances under continuous frying conditions. The results were compared with that of palm olein (PO). The ratio of the oil blends were: 60:40 (PO/SFO), 70:30 (PO/CNO) and 50:50 (PO/CSO). Potato chips were fried at 180 °C for a total of 56 h of operation. Analytical parameters such as tocols content, induction period, colour, free fatty acid, smoke point, primary and secondary oxidation values, polar compounds and polymer compounds were evaluated over the frying period. Blending PO with unsaturated oils was generally proved to keep most qualitative parameters comparable to those obtained in PO. In fact, none of the oils exceeded the legislative limits for used frying oils, *i.e.*, 0.5% for free fatty acids, 25% for polar compounds and 10% for polymer compounds. In the case of smoke point, all values were well above the end point of 170 °C. The concentration of tocols was satisfactorily retained in the four oils over the period of frying operation. It was also noted that blending PO with SFO gave higher resistance towards oxidative and hydrolytic behaviours as compared to other oil blends. The data obtained from the study would benefit food manufacturers who are searching for stable oils, particularly for use in industrial frying, without the need to fully replace their preference oils with palm olein (Ahmad Tarmizi A A; *JAOCS* 2008; 85: 245).

### 2.36. Crystal Size Distribution of Palm Oil and the Ternary Blend during Isothermal Crystallization Using Focused Beam Reflectance Measurement (FBRM) System—Zaliha Omar, Elina Hishamuddin, Norizzah Abdul Rashid

The aim of this study was to determine the effect of palm oil (PO) with palm stearin (PS) and palm olein (POo) on the crystal size distribution during isothermal crystallization. The crystal size distribution of refined, bleached and deodorized palm oil (PO) and the ternary blend of PO:PS:POo at weight ratio of 50, 30 and 20 (w/w) were monitored at 30 °C using a Focused Beam Reflectance Measurement (FBRM). Results showed that induction times (τ) of the blend and PO were 99 min and 110 min, respectively. The chord length distribution (CLD) of PO at the end of crystallisation process were 61 µm with smaller amount of crystals of 21 µm size, while most of the ternary blend crystals were 50 µm in size. These results were supported by the microscopy observation using Polarised Light Microscope. The different in the induction times and the CLD of PO and the ternary blend were possibly due to the chemical characteristics such as the triacylglycerol composition and fatty acid profile of each type of oils in the blend. The ternary blend nucleated at faster rate to form smaller crystal as compared to PO. The faster nucleation rate could be due to the higher content of high melting TAGs of the blend such as PPP (9.5%) and PPS (2.51%) as compared to PO (PPP 3.82% and PPS 1.39%), which also caused the blend slurry denser than PO. In conclusion, PS and POo altered the crystal size distribution of PO (Hishamuddin E *J. Crystal Growth* 2011; 335: 172).

### 2.37. Genetic and Environmental Effects on Canola Oil Quality Traits, Tocopherols and Carotenoids—Clare Flakelar, David Luckett, Julia Howitt, Greg Doran Paul D. Prenzler

Canola is an important oil crop for farmers in southern Australia and contributes millions of dollars in agricultural and food-sector earnings. New varieties are needed to address many factors, one of which is the “development of new export markets and uses for Australian oilseeds and products including new health and functionality oils” (Australian Oilseeds Federation Strategic Plan). Tocopherols and carotenoids are two classes of compounds that may confer health-enhancing properties and functionality to canola oil, yet few studies have been undertaken to simultaneously quantify these compounds in Australian canola varieties. The present study investigated 156 canola oil samples comprising 28 varieties collected from 14 different locations in southern NSW. Standard oil quality parameters were assessed along with quantitation of α-, γ-, and δ-tocopherol, β-carotene and lutein. Results will be presented on correlations among the various quantities measured as well as a REML analysis of G × E effects. The latter analysis showed that the major effect on levels of tocopherols and carotenoids was variety, opening up the possibility of breeding programs targeting genotypes with enhanced levels of these compounds.

### 2.38. Purification of Alaskan Walleye Pollock (*Theragra chalcogramma*) and New Zealand hoki (*Macruronus novaezelandiae*) Liver Oil Using Short Path Distillation—Matthew R. Miller, Alexandra C.M. Oliveira

The beneficial health effects of a diet rich in omega-3 long chain polyunsaturated fatty acids (*n*-3 LC-PUFAs) have been fully described in recent years. Marine oils are an important dietary source of *n*-3 LC-PUFAs, being especially rich in two of the most important fatty acids of this class, EPA (eicosapentaenoic acid; 20:5ω3) and DHA (docosahexaenoic acid; 22:6ω3). Because of its nutritional value, there is growing interest in refining fish oil for human consumption; however, the highly unsaturated *n*-3 LC-PUFAs are prone to oxidation, leading to oil rancidity. New Zealand’s hoki (*Macruronus novaezelandiae*) and the Alaskan pollock (*Theragra chalcogramma*) are major and important fisheries of their respective countries. Both produce large quantities of fishery byproducts, in particular crude or unrefined *n*-3 LC-PUFAs oils. Presently these oils are used as ingredients for animal feed, and only small amounts are used as human nutritional products. The aim of this research was to investigate the applicability of short path distillation for the purification of pollock and hoki oil to produce purified human-grade fish oil to meet quality specifications. Pollock and hoki oils were subjected to short path distillation and a significant decrease in free fatty acids and lipid oxidation (peroxide and anisidine values) products was observed. As a result, purified oils met the GOED standard for edible fish oils. The main advantages of using this technology, compared with traditional fish oil purification steps, are that it reduces the use of chemicals during processing and subsequent effluent discharge volumes, and it decreases the number of steps needed to refine fish oils.

### 2.39. Oxidation Stability and Antioxidant Activities of *Moringa oleifera* Seed Oil Extracted Using Cold Screw Press and Solvent Methods—Porjai Thamakorn and Pratumporn Chatthai

Oil was extracted from *Moringa oleifera* seed collected from organic farming community enterprise, Lopburi province, Thailand, and was determined following extraction with cold screw press (CSP), soxhlet extraction (SE), and cold solvent extraction (CSE), hexane was used as solvent, oil yield obtained was 10.02%, 34.99%, and 22.04% respectively. In measuring oxidation stability using Rancimat method in term of oxidative stability index (OSI), CSE oil had the highest stability followed by SE and CSP with values of 47.37 ± 0.52 h, 42.70 ± 1.33 h, and 25.90 ± 3.68 h respectively. Total phenolic compounds (µg Gallic acid Equivalent mL^−1^) was investigated, CSE oil get the maximum content of 173.75 ± 0.63 whereas SE and CSP contained 167.86 ± 0.11 and 163.65 ± 0.70 respectively. A test measuring the ferric reducing ability of plasma, the FRAP assay (µg Trolox Equivalent mL^−1^) for assessing antioxidant power showed again maximum in CSE (3.57 ± 0.14) followed by SE (2.80 ± 0.13) and CSE (2.52 ± 0.02) was the lowest. The free radical scavenging activity of oil samples against by DPPH (µg Trolox Equivalent mL^−1^) was determined with values of 646.28 ± 0.18, 474.01 ± 0.08, 442.98 ± 0.06 for CSE, SE, and CSP respectively. This study indicates that the extraction method was found to influence the relative amount of principle compounds and reflect to the oil quality. (Abdulkarim SM *Food Chem.* 2005; 93: 253). 

### 2.40. Revisiting the Thiobarbituric Acid Reactive Substances (TBARS) Assay to Measure Antioxidant Activity in a Lipid System—Md Ahsan Ghani, Celia Barril, Danny R. Bedgood, Paul D. Prenzler

There are many widely used methods to measure antioxidant activity (e.g., ORAC, TEAC, FRAP, DPPH), but very few assays measure the effectiveness of an antioxidant as it protects a lipid substrate. One test that has been used in this way is the thiobarbituric acid reactive substances (TBARS) assay, but it has been reported to be highly variable (Buenger J *Int. J. Cosmet. Sci.* 2006; 28: 135). Variability may arise from the substrate, the antioxidant, and even the order of addition of the reagents. Using linoleic acid as the substrate, TBARS assays exhibited variability in the absence of an antioxidant, thereby indicating possible batch-to-batch differences in the substrate itself. Within a batch of linoleic acid, different Trolox concentrations gave different %CV with the highest %CV at 1000 µM Trolox. The order of addition of the reagents was also found to affect the consistency of the results. To our knowledge, this is the first systematic study of the TBARS antioxidant assay specifically investigating method variability. Several sources of variability have been identified. It is possible that batch-to-batch variation in linoleic acid could be overcome by reporting % inhibition, if other sources of variability (e.g., antioxidant concentration) can be understood. Ongoing research in this area is investigating lipid oxidation markers other than TBARS (e.g., hydroperoxides) to determine if these can be used to more reliably assess antioxidant activity.

### 2.41. Enhancing a Niche, Sustainable, High-Value Omega-3 Source—Greenshell™ Mussel (*Perna canaliculus*)—Matthew R. Miller, Zoe Hilton, Luke Pearce, Bodhi Bettjeman and Nigel Joyce

Greenshell™ mussels (GSM—*Perna canaliculus*) are a sustainable source of omega-3 long chain polyunsaturated acids (*n*-3 LC PUFA). They are considered sustainable as they are farmed in New Zealand and Chile with dietary inputs. GSM derive their important omega-3 oil content from filter feeding on the source marine microalgae. GSM oil is a niche high-value product, with small volumes (estimated 5 tonnes in 2012) sold for high prices (estimated 1000× the cost of fish oils). GSM oil also contains a series of minor lipid components (non-methylene interrupted (NMI)-FA, plasmalogen, phytosterols and furan fatty acids) that are not contained in most fish oil products and that have been shown to have their own unique beneficial properties. There is a growing body of evidence that GSM oil may have its own unique health benefits, in particular in relation to reducing inflammation. The high cost of GSM oils is associated with low yields and the extraction technologies such as supercritical CO_2_ that are used to prepare the products. Because of the increasing cost and limited availability of traditional fish oils, along with the growing demand for carnivorous aquaculture species such as salmon, there is scope for enhancement of novel and sustainable omega-3 rich sources. This study is determining the potential for GSM as a local sustainable source of omega-3 and is identifying where the organism stores its *n*-3 LC PUFA. This work aims to enhance the small NZ GSM oil industry by improving quality and yield of the oil.

### 2.42. Unusual Sterols in Infant Food Products Supplemented with PUFAs from Single-Cell Oils—Jacqui L. Adcock, Colin J. Barrow

Long-chain polyunsaturated fatty acids (LCPUFAs) such as docosahexaenoic acid (DHA) and arachidonic acid (ARA) are important to the growth and development of infants. Endogenous synthesis of DHA and ARA alone may not meet the demands of the developing tissues of infants (especially pre-term infants) and therefore, a dietary source is also beneficial. DHA and ARA are naturally found in breast milk and are often supplemented in infant formulas to mimic the composition of natural breast milk. The three main sources of LCPUFAs added to infant formulas are fish oils, egg yolk lipids and oils from single-cell organisms (single-cell oils, SCO) such as algae and fungi. The advantage of single-cell oils is that they can be produced with relatively high concentrations of DHA or ARA, but without other (possibly undesirable) LCPUFAs, allowing for more control over the composition of the formula. They also do not rely on declining fish stocks and are considered a vegetarian source of LCPUFAs. Microorganisms are known to contain a wide range of sterol structures, both common and rare (Volkman JK, *Appl. Microbiol. Biotechnol.* 2003; 60: 495). In this study, we examine the sterol profiles of infant formula and yoghurt supplemented with single-cell oils. The oils were extracted from the foods, saponified and the sterol fraction isolated. The sterols were then converted to trimethylsilyl esters prior to analysis by GC-MS. We detected at least twelve different sterols in the infant formula and ten in the yoghurt. These sterol profiles were compared to those of a number of single-cell oils.

### 2.43. Lysophosphatidic Acid Acyltransferases (LPAATs) from *Ricinus communis* with Specific Activity towards Ricinoleate—Shoko Okada, Craig Wood, Surinder Singh, Xue-Rong Zhou

Ricinoleic acid (RA) is an industrially important fatty acid that is produced in developing seeds of several plants including castor *Ricinus communis*, where it accumulates up to 90% in the oil. A previous study on transgenic plants expressing the castor oleate hydroxylase indicated that the bottleneck to higher accumulation of RA was in part due to the lack of a lysophosphatidic acid acyltransferase (LPAAT) that could incorporate RA into the s*n*-2 position of the glycerol backbone (Bates PD *Plant J.* 2011; 68: 387). We investigated four putative castor LPAAT genes known to be expressed in the seed during development for their ability to incorporate RA into oil. Using a *Nicotiana benthamiana* leaf transient expression system and subsequent *in vitro* assay using radiolabeled glycerol-3-phosphate and acyl-coenzyme A as substrates we identified two castor LPAATs that produced dioleoylphosphatidic acid from oleoyl-coenzyme A. These two castor LPAATs also produced diricinoleoylphosphatidic acid from ricinoleoyl-coenzyme A, with one LPAAT (LPAAT2) having significantly higher acyl transfer activity than the other. When the two castor LPAATs were supplied with equimolar amounts of oleoyl- and ricinoleoyl-coenzyme A the assay containing LPAAT2 was able to produce monoricinoleoyl triacylglycerol with the aid of the endogenous triacylglycerol assembly machinery of *N. benthamiana*. Use of these two castor LPAATs may further increase RA accumulation in transgenic plant systems.

### 2.44. The Assessment of Australian Canola Oil for Relationships between Oxidative Stability, Trace Elements and Fatty Acid Profiles of Selected Cultivars—Clare Flakelar, Julia Howitt, Greg Doran, David Luckett, Paul D. Prenzler

The growing of canola in Australia has become a major agricultural practice. The assessment of minor constituents in the canola oil and its quality parameters with regard to Australian canola varieties has not been researched in great depth. This study implements numerous analytical techniques to quantify and assess a sample set of 30 different Australian varieties collected from 14 growing locations across Southern NSW. During this work, an optimised method for the microwave digestion of samples for trace element analysis was developed. Analyses involved the use of Rancimat (oxidative stability), Inductively Coupled Plasma Mass Spectroscopy (ICP-MS) (trace elements) and Gas Chromatography (GC) (fatty acid profile) methods. Statistically significant relationships between oxidative stability, trace elements and fatty acid profile will be reported with respect to seed variety and growing location. These results form part of a larger project consisting of the method development and quantification of numerous minor components in canola oil providing baseline data for development of new canola lines.

### 2.45. Health Effects of Long Chain Omega-3 Fatty Acids in People with Chronic Obstructive Pulmonary Disease: A Systematic Review—Ashley Fulton, Alison Hill, Marie Williams, Peter Howe, Alison Coates

This systematic review investigated relationships between long chain omega-3 polyunsaturated fatty acids (LC-*n*3PUFAs) and health indicators in people with chronic obstructive pulmonary disease (COPD), including methods used to quantify LC*n*-3PUFA. Studies were eligible for inclusion if they were experimental or observational studies in adults with COPD, included assessments of LC-*n*3PUFAs and published in English. Medline, Embase, AMED, Scopus, Web of Science, Cochrane library, CINAHL, Informit health databases and the WHO international clinical trial registry were searched in late June to early July 2013. Additional studies were identified through reference lists of included studies and database alerts. Search terms included obstructive lung disease, chronic obstructive pulmonary disease (COPD), emphysema, chronic bronchitis, COAD, COBD, AECB, omega-3 fatty acid, omega-6 fatty acid, (essential) fatty acid, omega-3, omega-6, polyunsaturated, docosahexaenoic acid (DHA), eicosapentaenoic acid (EPA), docosapentaenoic acid and fish oil. Two independent investigators confirmed the search strategy, screening of abstracts for inclusion and data extraction. Of the 2073 citations returned full text articles were retrieved for 43, with 11 observational studies retained for the review. Great heterogeneity existed between studies in terms of criteria for and severity of COPD, assessment of LC*n*-3PUFA dietary intake or content of plasma phospholipids or erythrocytes and the choice of health indicators. Preliminary results indicate a poverty of studies exploring relationships between LC-*n*3PUFAs and COPD. In those available there is conflicting evidence for both the relationship between LC*n*-3PUFAs and COPD and potential health benefits in people with COPD.

### 2.46. Characterization of the Lipid Class and Fatty Acid Composition of DHA-Containing Camelina Sativa Oilseed—Maged P. Mansour, Pushkar Shrestha, Srinivas Belide, James R. Petrie, Surinder P. Singh, Peter D. Nichols

The lipid content of DHA-Camelina seeds (36% w/w) was mainly triacylglycerols (TAG 86%). Most of the lipid (31% w/w) was extracted by hexane. A subsequent chloroform-methanol (CM) extraction recovered a further 4.8% of polar lipid (PL) rich extract, while the residual lipid released by transmethylation of the extracted meal accounted for 0.3% (w/w). The hexane extract was TAG rich (96%) with some residual TAG in the CM extract (44%). The main phospholipid species was phosphatidyl choline (69%), followed by phosphatidyl ethanolamine (13%). The seeds contained 6.8% DHA in the TAG hexane fraction, 4.2% in the PL rich CM extract, 6.1% in the residual TAG in the PL rich CM fraction, 3.0% in the glycolipid fraction and 1.6% in the phospholipid fraction. Levels of ALA were higher than in control seeds (39%–54% *vs.* 12%–32%). The main sterols were: sitosterol (43%–54%), campesterol (21%–26%), cholesterol (5%–8%), brassicasterol (2%–7%) and isofucosterol (4%–6%). 24-dehydrocholesterol, cholest-7-e*n*-3-ol, campestanol and stigmasterol were minor components. Several fatty alcohols were detected including uncommon iso-branched and odd-chain components: iso-17:0 (16%–38%), 17:0 (0.3%–6%), 19:0 (5%–7%), 16:0 (6%–12%), iso-18:0 (7%–14%), 18:0 (6%–9%) and 18:1 (3%–5%). To our knowledge, the presence of high levels of iso branched odd-chain fatty alcohols is a novel finding and these components could be derived from wax esters, sterol esters or free fatty alcohols. The lipid class and FA profiles of DHA-containing Camelina sativa oilseeds may provide key information for oil chemists and other researchers seeking new sustainable sources of these health-benefitting long-chain omega-3 oils.

### 2.47. Evaluating the Use of Pretreated Cellulosic Biomass for the Microbial Production of Biofuels, Lipids and Carotenoids—Reinu E. Abraham, Adarsha Gupta, Colin J. Barrow, Munish Puri

The availability of large quantities of lignocellulosic feedstock ranging from woody biomass (softwood and hardwood) to grasses makes Australia a great reservoir for the production of fermentable sugars. The utilisation of this locally available wood waste through bioprocessing to produce high yields of fermentable sugars is valuable in biofuel production. Sugar hydrolysate from woody biomass is a potential carbon source for growing lipid producing marine microbes, for use in biofuel, carotenoid and omega-3 production. At Deakin University, we have developed methods for producing sugar hydrolysate from locally available low cost cellulosic biomass. This study aims at producing reducing sugar hydrolysate from pretreated biomass (*Cannabis sativa*) and its use by marine microbes for producing ethanol. Industrial hemp was obtained as a cellulosic waste to produce fermentable sugars from pretreated biomass. Quantification of reducing sugars was achieved using high performance liquid chromatography (HPLC). The profile of total lipids and unsaturated fatty acid produced from novel marine microbes was documented by gas chromatography (GC). The presentation will focus on the results obtained from this study, including optimised cellulosic pretreatment methods (Abraham RE *et al.*, Biomass Bioenergy; 2013; 58: 180–187), reducing sugar production and its scale up, and the utilisation of feed material for producing omega-3 oils and carotenoids.

### 2.48. Synthetic Biology Based Engineering Improves Lipid Accumulation and PUFA Biosynthesis in Budding Yeast—Shailendra P. Sonkar, Munish Puri and Colin J. Barrow

The market demand for polyunsaturated fatty acid (PUFA) is high due to its role in maintaining good health and the prevention of cardiovascular, diabetes and arthritis related modern age diseases. (Adarme-Vega T.C. *Curr. Opin. Biotechnol.* 2014; 26: 14). However, limited natural resources and continuous increasing global demand of PUFA as functional food ingredients and nutritional supplements requires the development of alternative sources, including microbial cell factory systems. In this study we investigated the potential of *Saccharomyces cerevisiae* to produce PUFA by exploiting synthetic biology and metabolic engineering tools. The codon optimized synthetic genes such as Diacylglycerol acyl transferase (*DGA1*), Acyl CoA synthase (*FAA3*), Delta 6 elongase, Delta 9, 12, 6, 5 and 17 desaturases were selected to clone under Gal1 and 10 promoters in yeast based dual expression vector. Genetically modified yeast were grown on various carbon sources in a nitrogen limited synthetic medium at 30 °C for fatty acid analysis. Results revealed an increase in lipid accumulation and biosynthesis of endogenous fatty acid after expression of *DGA1* and *FAA3* genes. Significant conversion of stearic acid (18:0, SA) to oleic acid (18:1, OA) was observed. Further, expression of *D12D* converted OA into linoleic acid (C18:2n6, LA) and α-linoleic acid (C18:3n3, ALA), and these conversions were optimised. In conclusion, engineered yeast showed increased lipid accumulation and biosynthesis of linoleic and alpha linoleic acid without supplying exogenous fatty acid in growth media. Further studies are in progress to expand pathway to synthesise omega-3 fatty acids.

### 2.49. Dietary Long Chain *n*-3 Polyunsaturated Fatty Acids Rduce Muscle Fatigue in Rats Independently of Cardiac Function—Renee Henry, Gregory E. Peoples, Peter L. McLennan

Fish oil (FO) feeding increases myocardial and skeletal muscle oxygen-use efficiency in association with long chain omega-3 polyunsaturated fatty acid (LC*n*-3PUFA) docosahexaenoic acid (DHA; 22:6*n*-3) incorporation into muscle cellular membranes. Physiological outcomes in rats are typically reported for diets containing 5%–10% FO, yet muscle fatty acid composition is responsive to extremely small intakes. This study tested the hypothesis that FO, in an achievable human dietary range, can modulate skeletal muscle fatigue in the rat. Male Wistar rats were fed for 5 w, a 10% fat diet containing: olive oil alone (control); 0.31% (lowFO); or 1.25% moderate (modFO) NuMega, High-DHA Tuna Fish Oil in olive oil. Isometric contractions of the gastrocnemius-plantaris-soleus muscle bundle were assessed in response to sciatic nerve stimulation using the constant-flow autologous blood-perfused hindlimb *in vivo*, with artificial ventilation ensuring optimal oxygenation. Hindlimb contractions of FO rats developed greater force throughout 2 Hz stimulation with significantly less decline over 5 min (control −80 ± 5%; lowFO −70 ± 3.7%; modFO −70 ± 3.6%). During 5 min repeat 5 s bursts of 5Hz stimulation, contractile force was greater with FO feeding and sustained for more contractions (time to decline 50%: control 73 ± 10 s; lowFO 118 ± 14 s; modFO 126 ± 14 s). In rats fed very low supplemental FO, contracting skeletal muscle *in vivo* was fatigue-resistant over a range of protocols, especially in the early aerobic metabolism phase, similar to exercise training and independent of heart rate and cardiac output. These results have applied-physiology implications for dietary LC*n*-3PUFA limiting fatigue symptoms and improving daily living activities in heart failure and other chronic conditions.

### 2.50. Fish Oil Inhibits Cardiac Hypertrophy in the Rat with very Low Supplemental Intakes of Long Chain *n*-3 PUFA—Renee Henry, Gregory E. Peoples, Peter L. McLennan

Extending well documented cardioprotective effects of marine-derived long chain omega-3 polyunsaturated fatty acids (LC*n*-3PUFA) against arrhythmia and sudden cardiac death, there is evidence for a role in preventing heart failure. We investigated the ability of fish oil (FO) feeding to prevent or reverse hypertrophic remodelling, using a low (human diet equivalent) intake of LC*n*-3PUFA. Male Wistar rats were fed diets containing 10% fat (22% energy) as either olive oil alone (control) or fish oil (0.3% tuna fish oil + 9.7% OO). The FO intervention was initiated either 4w before or 1w after aortic banding surgery to produce pressure overload and cardiac hypertrophy. Heart weight index and myocardial fatty acid composition was measured 5 w post-surgery. Dietary FO increased myocardial phospholipid docosahexaenoic acid (DHA 22:6*n*-3: control 7%; FO 18%) and reduced arachidonic acid (20:4*n*-6: control 23%, FO 18%). A 20% increase in heart weight index occurred in control hearts, prevented by fish oil feeding when provided 4w before (+2%, *P* = 0.80) but not 1w after pressure-overload (+15% *P* = 0.041). Dietary FO reduced heart rate by 10% and increased stroke volume by 40% in non-hypertrophied rat hearts. This study showed that a (non-therapeutic) FO diet was effective in preventing cardiac remodelling but only if given prior to the hypertrophic stimulus, and demonstrated that physiological effects can be observed with small, nutritionally relevant intakes of LC*n*-3PUFA. Given this study’s energy-equivalent daily dose of ~570 mg EPA + DHA for a 70 kg man, the results provide a basis for linking animal studies to human physiology, pathophysiology and epidemiology.

### 2.51. Increasing Triacylglycerol (TAG) Content in Leaves via Monoacylglycerol Acyl Transferase (MGAT) Pathway can Induce Senescence in *Nicotiana benthamiana*—Uday K. Divi, Anna El Tahchy, Thomas Vanhercke, James R. Petrie, and Surinder P. Singh

Approaches to enhance triacylglycerol (TAG) content in plant vegetative parts have gained significant attention for potential bio-fuel applications. In an attempt to increase TAG levels in leaves, we previously demonstrated that a novel substrate, monoacylglycerol (MAG) can be used for the biosynthesis of diacylglycerol (DAG) and TAG via the MGAT pathway. Transient expression of *Mus musculus* *MGAT*s (*MGAT1 or 2*) in model plant *Nicotiana benthamiana* increased TAG levels at 5 days post infiltration. Here we show that increased TAG and DAG levels can be achieved by as early as 2 dpi. In addition, the *MGAT1* infiltrated leaf areas showed visible senescence from 3 dpi. In order to understand the molecular mechanism underlying TAG increase and senescence *de* *novo* assembling and annotation of *N. benthamiana* leaf transcriptome was carried out via Illumina deep sequencing. The *MGAT1* responsive transcriptome was identified and characterized. We found that *MGAT1* responsive genes affect several processes including TAG biosynthesis, photosynthesis, cell-wall, cutin, suberin, wax and mucilage biosynthesis, lipid and hormone metabolism. Comparative analysis with other senescence related studies revealed significant overlap in transcriptional responses. Understanding the molecular and biochemical link between *MGAT1*-induced TAG accumulation and senescence in the leaves should help in developing strategies to increase TAG content of leaves via the MGAT pathway.

### 2.52. Dietary Fish Oil Improves Skeletal Muscle Oxygen Efficiency in Healthy Trained Males under Fatigued Stress—Lachlan J. Hingley, Michael J. Macartney, Marc Brown, Peter L. McLennan, Gregory E. Peoples

Skeletal muscle membrane fatty acid composition alters in response to the dietary provision of long chain omega-3 polyunsaturated fatty acids (LC*n*-3PUFA), particularly docosahexaenoic acid (DHA). In rats fed fish oil, muscle fatigue is attenuated as represented by sustained muscle contractile force coupled with improved oxygen (O_2_) efficiency. Enhanced O_2_ efficiency is also supported in humans, however, study design issues have limited muscle fatigue observations. Therefore we tested if a dietary achievable dose of fish oil could improve muscle contractile performance as a result of improved O_2_ efficiency. A double blind matched design involved twenty eight physically fit male participants supplemented with (2 × 1 g/day) soy oil (Control) or high DHA tuna fish oil (NuMega) (FO), delivering daily: DHA 560mg and eicosapentaenoic acid (EPA) 140 mg, for eight weeks. Using a cycle ergometer, O_2_ consumption and work capacity were collected under conditions of sub-maximal exercise, supra maximal repeat bout exercise (6 × 30 s)/recovery and cycling time trial (5 min). In addition, isometric MVC of the quadriceps muscle was measured pre/post cycling to represent conditions of fatigue. Work capacity during submaximal, supramaximal repeat bout sprinting (Control: 545 ± 28; FO: 511 ± 33 W) or fatigued time trial (Control: 267 ± 19; FO: 253 ± 16 W) were not affected by FO. Equally, MVC either in an unfatigued or fatigued state (Control: 270 ± 66; FO: 251 ± 69 N) did not change. However, FO supplementation improved oxygen efficiency (Control: −23 ± 26; FO: −154 ± 59 mL O_2_/min/100W *P* < 0.05). This study demonstrates that dietary achievable dosage LC*n*-3PUFA improves oxygen efficiency during stressful exercise against a background of high physical fitness in healthy human males. 

### 2.53. Mass Spectrometry Imaging of Lipid Distribution in Transgenic Tobacco Leaves with Enhanced Oil Accumulation—Qing Liu, Thomas Vanhercke, Anna El Tahchy, Xue-Rong Zhou, Pushkar Shrestha, Uday K. Divi, Christopher N. James, Patrick J. Horn, Kent D. Chapman, James R. Petrie, Surinder P. Singh

Storage lipids, mainly triacylglycerols (TAG), don’t normally accumulate in plant vegetative tissues in large amounts. A high oil transgenic tobacco (*Nicotiana tabacum* L.) line that we recently generated ectopically expressing *WRI1, DGAT1* and *OLEOSIN* genes was able to produce more than 15% TAG in mature leaves. Lipid droplets were visualized by epifluorescence microscopy using neutral lipid-specific dye, such as BODIPY. Using the matrix-assisted laser desorption/ionization mass spectrometry imaging (MALDI-MSI) system, we have made a comparative study on the spatial distribution of TAG species as well as phospholipids and chloroplast galactolipids between the high oil and wild type tobacco leaves. Cross sections of a tobacco leaf adhered to a glass slide were coated with matrix that was co-crystallized with the analytes prior to scanning. On each particular *x*,*y* position of the selected cross section, lipid ions as [M + H]^+^, [M + Na]^+^, and [M + K]^+^ adducts were generated by laser beam and fed to the Orbitrap mass analyser that collects mass spectra consecutively over the entire sample section, which were used to generate a MS image. Analysis of the spatial distribution of major TAG species, such as 52:3 and 52:4 confirmed that elevated TAG species were distributed throughout the leaf mesophyll of the transgenic tobacco leaf. There was a clear raise of DAG and TAG species containing one or more palmitic acid (C16:0) and oleic acid (C18:1) at the expense of those containing α-linolenic acid (C18:3). Similarly, the raise of PC species, such as 34:1 at the expense of 36:5 and 36:6 was observed in transgenic *vs.* WT leaves. In the main chloroplast galactolipids, such as monogalactosyldiacylglycerol (MGDG), significantly higher levels of 36:4 and 36:5 species and lower level of 36:6 were observed in the transgenic line compared to WT tobacco. Further, the average compositions of molecular species visualized in MALDI-MSI were generally in line with the Q-TOF LC-MS results, demonstrating the validity and suitability of applying such a cutting edge technology.

## 3. Author Affiliations

- Adam Ismail, Global Organization for EPA and DHA Omega-3, Salt Lake City, UT, USA
- Adarsha Gupta, Deakin University, Australia
- Alexandra C.M. Oliveira, Kodiak Seafood and Marine Science Centre, University of Alaska Fairbanks, USA
- Alison Coates, The University of South Australia, Australia
- Alison Hill, The University of South Australia, Australia
- Allan Green, CSIRO Plant Industry, Australia
- Amani Alhazmi, School of Health Sciences, University of Newcastle, Australia
- Andreas Menzel, Cargill Global Food Research, Belgium
- Andrew MacKenzie, Callaghan Innovation Ltd., Lower Hutt, New Zealand
- Andrew Pipingas, Swinburne University of Technology, Australia
- Anita Lee, Royal Adelaide Hospital, Australia
- Anna El Tahchy, CSIRO Food Futures National Research Flagship, Canberra, ACT, Australia
- Anne-Marie Minihane, University of East Anglia, Norwich, United Kingdom
- Ashley Fulton, The University of South Australia, Australia
- Azmil Haizam Ahmad Tarmizi, Malaysian Palm Oil Board, Malaysia
- Barbara Meyer, University of Wollongong, Australia
- Bev Muhlhausler, FOODplus Research Centre, The University of Adelaide, Australia
- Bodhi Bettjeman, New Zealand Institute for Plant and Food Research Limited, New Zealand
- Bruce Smallfield, New Zealand Institute for Plant and Food Research, New Zealand
- Cathryn A. Conlon. Massey University, Auckland, New Zealand
- Celia Barril, School of Agricultural and Wine Sciences, Charles Sturt University, Australia
- Christopher N. James, Department of Biological Sciences, University of North Texas, USA
- Cindy Hall, Royal Adelaide Hospital, Australia
- Cintia B. Dias, University of Newcastle, Australia
- Clare Flakelar, School of Agricultural and Wine Sciences, Charles Sturt University, Wagga Wagga and Graham Centre for Agricultural Innovation, Australia
- Clemens von Schacky, Preventive Cardiology, University of Munich and Omegametrix, Martinsried, Germany
- Colin J. Barrow, Centre for Chemistry and Biotechnology, Deakin University, Australia
- Craig Wood, CSIRO Plant Industry, Australia
- Crystal Haskell, David Kennedy. Northumbria University, Newcastle, UK
- Danny R. Bedgood, School of Agricultural and Wine Sciences, EH Graham Centre for Agricultural Innovation, Charles Sturt University, Australia
- DanYang Ying, CSIRO Animal, Food and Health Sciences, Australia
- David Colquhoun, University of Queensland, Wesley, Australia
- David Luckett, NSW Department of Primary Industries, Australia
- Elina Hishamuddin, Malaysian Palm Oil Board (MPOB), Malaysia
- Elizabeth Stojanovski, School of Mathematical and Physical Sciences, University of Newcastle, Australia
- Faizan Jameel, The University of Newcastle, Australia
- Frederick M. Pfeffer, Centre for Chemistry and Biotechnology, Deakin University, Waurn Ponds, Victoria, Australia
- Giovanni M. Turchini, Deakin University, Australia
- Greg Doran, School of Agricultural and Wine Sciences, Charles Sturt University, Wagga Wagga and Graham Centre for Agricultural Innovation, Australia
- Gregory E. Peoples, School of Health Sciences, University of Wollongong, Australia
- Hywel Griffiths Photonz, New Zealand
- Jacqui L. Adcock, Centre for Chemistry and Biotechnology, Deakin University, Australia
- Jagan Billikanti, Callaghan Innovation Ltd., Lower Hutt, New Zealand
- James R. Petrie, CSIRO Food Futures National Research Flagship and CSIRO Plant Industry, Canberra, Australia
- Jaroslav A. Kralovec, Ocean Nutrition Canada Ltd., Dartmouth, Nova Scotia, Canada
- Jeff I McCormick, Lincoln University, New Zealand
- John Podd, Stephen R Hill, Massey University, Palmerston North, New Zealand
- Jonathan D. Buckley, Nutritional Physiology Research Centre, University of South Australia, Australia
- Julia Howitt, School of Agricultural and Wine Sciences, Charles Sturt University, Wagga Wagga, Australia
- Julian Cribb, Julian Cribb and Associates, Australia
- Karen Charlton, University of Wollongong, Australia
- Kathrine Allikian, Photonz, New Zealand
- Kent D. Chapman, Department of Biological Sciences, Centre for Plant Lipid Research, University of North Texas, USA
- Lachlan J. Hingley, School of Health Sciences, University of Wollongong, Australia.
- Leah McWilliams Royal Adelaide Hospital, Australia
- Leslie G. Cleland, Rheumatology Unit, Royal Adelaide Hospital, Australia
- LiJiang Cheng, CSIRO Animal, Food and Health Sciences, Australia
- Lisa G. Wood. School of Biomedical Science and Pharmacy, University of Newcastle, Australia
- Llew Spargo, Royal Adelaide Hospital, Australia
- Luke Pearce, The New Zealand Institute for Plant and Food Research Limited, New Zealand
- Luz Sanguansri, CSIRO Animal, Food and Health Sciences, Australia
- Maged P. Mansour, CSIRO Marine and Atmospheric Research, Hobart, Australia
- Manohar L. Garg, School of Biomedical Sciences and Pharmacy, University of Newcastle, Australia
- Marc Brown, School of Health Sciences, University of Wollongong, Australia
- Marie Williams, The University of South Australia, Australia
- Mark Dines, Photonz, New Zealand
- Mark McEvoy, Centre for Clinical Epidemiology and Biostatistics, University of Newcastle, Australia
- Mary Ann Augustin, CSIRO Animal, Food and Health Sciences, Australia
- Matt Taylor, CSIRO Ecosystem Sciences, Australia
- Matthew R. Miller, New Zealand Institute for Plant and Food Research Limited, New Zealand
- Md Ahsan Ghani, School of Agricultural and Wine Sciences, EH Graham Centre for Agricultural Innovation, Charles Sturt University, Australia
- Melinda Phang, The University of Newcastle, Australia
- Melissa K. Gregory, Rheumatology Unit, Royal Adelaide Hospital, Australia
- Michael J. Macartney, School of Health Sciences, University of Wollongong, Australia
- Michael J. James, Rheumatology Unit, Royal Adelaide Hospital, Australia
- Moreland Gibbs, Photonz, New Zealand
- Munish Puri, Deakin University, Australia
- Nick Goddard, Australian oilseed federation, Australia
- Nigel Joyce, The New Zealand Institute for Plant and Food Research Limited, New Zealand
- Nils Hoem, Aker BioMarine, Oslo, Norway
- Norizzah Abdul Rashid, Universiti Teknologi MARA, Shah Alam, Selangor, Malaysia
- Owen Catchpole, Callaghan Innovation Ltd., Lower Hutt, New Zealand
- Patrick J. Horn, Department of Biological Sciences, Centre for Plant Lipid Research, University of North Texas, USA
- Paul D. Prenzler, School of Agricultural and Wine Sciences, EH Graham Centre for Agricultural Innovation, Charles Sturt University, Australia
- Paul Smith, Cargill Global Food Research, Belgium
- Peter D. Nichols, CSIRO Food Futures Flagship and CSIRO Marine and Atmospheric Research, Hobart, Australia
- Peter Dyer, Callaghan Innovation Ltd., Lower Hutt, New Zealand
- Peter Howe, The University of Newcastle, Australia
- Peter L. McLennan, Graduate School of Medicine, University of Wollongong, Australia
- Peter Tait, Lincoln University, New Zealand
- Philippa Lyons, Wall. Edith Cowan University, Australia
- Polly Dobson, Centre for Chemistry and Biotechnology, Deakin University, Geelong, Australia
- Porjai Thamakorn, Faculty of Agro-Industry, King Mongkut’s Institute of Technology Ladkrabang, Bangkok, Thailand
- Prakash Adhikari, Cargill Global Food Research, China
- Pratumporn Chatthai, Faculty of Agro-Industry, King Mongkut’s Institute of Technology Ladkrabang, Bangkok, Thailand
- Pushkar Shrestha, CSIRO Food Futures National Research Flagship, Canberra, Australia
- Qing Liu, CSIRO Food Futures National Research Flagship, Canberra, Australia
- Rahau Shirazi, Callaghan Innovation, Australia
- Razali Ismail, Malaysian Palm Oil Board, Malaysia
- Reinu E. Abraham, Deakin University, Australia
- Renee Henry, School of Health Sciences, University of Wollongong, Australia
- Sean Smith, Cargill Global Food Research, USA
- Setyaningrum Rahmawaty, University of Wollongong, Australia
- Shailendra P. Sonkar, Deakin University, Australia
- Shoko Okada, CSIRO Ecosystem Sciences, Australia
- Srinivas Belide, CSIRO Plant Industry, Canberra, Australia.
- Stephen Tallon, Callaghan Innovation Ltd, Lower Hutt, New Zealand
- Sukhdeep Bhail, CSIRO Animal, Food and Health Sciences, Australia
- Surinder P. Singh, CSIRO Food Futures National Research Flagship, Canberra, ACT, Australia
- Susan Marshall, Seafood Technologies, The New Zealand Institute for Plant and Food Research Limited, Nelson, New Zealand
- Susanna Proudman, Royal Adelaide Hospital and University of Adelaide, Australia
- Taiwo O. Akanbi, Centre for Chemistry and Biotechnology, Deakin University, Australia
- Thomas Vanhercke, CSIRO Food Futures National Research Flagship, Canberra, Australia
- Tim D. Nalder, Seafood Technologies, New Zealand Institute for Plant and Food Research Ltd., Nelson, NZ and Centre for Chemistry and Biotechnology, Deakin University, Australia
- Uday K. Divi, CSIRO Food Futures National Research Flagship, Canberra, ACT, Australia
- Vonny Fasi, Lincoln University, New Zealand
- Welma Stonehouse, CSIRO, Adelaide, Australia; Massey University, Auckland, New Zealand
- Xue-Rong Zhou, CSIRO Plant Industry, Canberra, Australia
- Zaliha Omar, Malaysian Palm Oil Board (MPOB), Malaysia
- Zhiping Shen, CSIRO Animal, Food and Health Sciences, Australia
- Zoe Hilton, Cawthron Institute, Australia
